# Stimulation Duration in Patients with Early Oocyte Maturation Triggering Criteria Does Not Impact IVF-ICSI Outcomes

**DOI:** 10.3390/jcm11092330

**Published:** 2022-04-22

**Authors:** Sophie Stout, Yohann Dabi, Charlotte Dupont, Lise Selleret, Cyril Touboul, Nathalie Chabbert-Buffet, Emile Daraï, Emmanuelle Mathieu d’Argent, Kamila Kolanska

**Affiliations:** 1 Service de Gynécologie Obstétrique et Médecine de la Reproduction, Hôpital Tenon, AP-HP, Sorbonne Université, 4 Rue de la Chine, 75020 Paris, France; sophie.sstout@gmail.com (S.S.); yohann.dabi@aphp.fr (Y.D.); lise.selleret@aphp.fr (L.S.); cyril.touboul@aphp.fr (C.T.); nathalie.chabbert-buffet@aphp.fr (N.C.-B.); emile.darai@aphp.fr (E.D.); emmanuelle.mathieu@aphp.fr (E.M.d.); 2 INSERM UMRS 938, Centre de Recherche Saint-Antoine, 27 Rue Chaligny, CEDEX 12, 75571 Paris, France; charlotte.dupont@aphp.fr; 3 Service de Biologie de la Reproduction-CECOS, Hôpital Tenon, AP-HP, Sorbonne Université, 4 Rue de la Chine, 75020 Paris, France

**Keywords:** stimulation duration, ovulation trigger, live-birth rate, pregnancy outcome, in vitro fertilisation

## Abstract

Results from studies reporting the optimal stimulation duration of IVF-ICSI cycles are inconsistent. The aim of this study was to determine whether, in the presence of early ovulation-triggering criteria, prolonged ovarian stimulation modified the chances of a live birth. This cross-sectional study included 312 women presenting triggering criteria beginning from D8 of ovarian stimulation. Among the 312 women included in the study, 135 were triggered for ovulation before D9 (D ≤ nine group) and 177 after D9 (D > nine group). The issues of fresh +/− frozen embryo transfers were taken into consideration. Cumulative clinical pregnancy and live-birth rates after fresh +/− frozen embryo transfers were similar in both groups (37% versus 46.9%, *p* = 0.10 and 19.3% versus 28.2%, *p* = 0.09, respectively). No patient characteristics were found to be predictive of a live birth depending on the day of ovulation trigger. Postponing of ovulation trigger did not impact pregnancy or live-birth rates in early responders. A patient’s clinical characteristics should not influence the decision process of ovulation trigger day in early responders. Further prospective studies should be conducted to support these findings.

## 1. Introduction

The criterion classically used in assisted reproductive technology (ART) to decide when to trigger oocyte maturation is the presence of a satisfying number of follicles (two or three) sized 16 to 22 mm [1]. However, as this decision may be influenced by other factors such as the size of the growing follicle cohort and hormonal levels on the day of monitoring, the European Society of Human Reproduction and Embryology (ESHRE) guidelines recommend a case-by-case approach [2]. The results from studies reporting optimal stimulation durations for patients undergoing in vitro fertilization (IVF) cycles are inconsistent [3,4,5,6]. Some authors have reported that women with a short duration of stimulation had the same likelihood of becoming pregnant as those with a longer duration of stimulation [3,4]. In contrast, Chuang et al. found that a prolonged duration of gonadotropin stimulation of 13 days or more was an independent negative predictive factor of ART success [5]. Sarka et al. recently reported that both prolonged and short stimulation durations were associated with lower live-birth rates, and that optimal outcomes were achieved for stimulation durations between 9 and 12 days [6].

However, all these studies analysed pregnancy outcomes independently of the day by which triggering criteria for final oocyte maturation were observed. Thus, the early presence of triggering criteria was not taken into account. In everyday practice, it is not uncommon to deal with early responders and it can be difficult to decide whether to prolong ovarian stimulation in this setting. The rapidity at which patients achieve criteria for ovulation triggering may vary depending on a number of factors such as ovarian reserve prior to stimulation, the cause of infertility, or the stimulation protocol. It has been reported for example that the antagonist protocol is associated with shorter stimulation durations [7].

The objective of this study was thus to determine whether a prolonged duration of ovarian stimulation in women with ovulation-triggering criteria achieved by day 7–8 of stimulation modifies the chances of a live birth. A second objective was to identify subgroups of patients for whom a longer stimulation duration was associated with a higher live-birth rate.

## 2. Materials and Methods

### 2.1. Patients

We conducted a retrospective analysis of a prospective database including women managed in the ART Unit of the Department of Gynaecology and Obstetrics of Tenon University Hospital, Paris (France) from December 2016 to July 2020. Among 3672 cycles of ovarian stimulation during the study period, we have selected all women undergoing a controlled ovarian stimulation (COS) for IVF/intracytoplasmic sperm injection-embryo transfer (IVF/ICSI-ET) (2771 cycles). Cancelled cycles have been excluded from the analysis. Among 2556 ovarian punctures for IVF/ICSI, criteria for triggering oocyte maturation by 7 or 8 days of treatment was achieved in 312 cycles. The criteria used to trigger oocyte maturation were the presence of at least three follicles >16 mm associated with oestrogen levels >400 pg/mL. We identified women for whom the decision to trigger ovulation was made as soon as the criteria were met (D ≤ 9 group), and those for whom treatments were prolonged by a few days aiming for better outcomes (D > 9 group). The decision on the day of ovulation trigger was made according to the ESHRE criteria which could have been adapted on an individual case basis depending on each practitioner’s experience and organizational factors in the ART unit.

**Figure 1:**
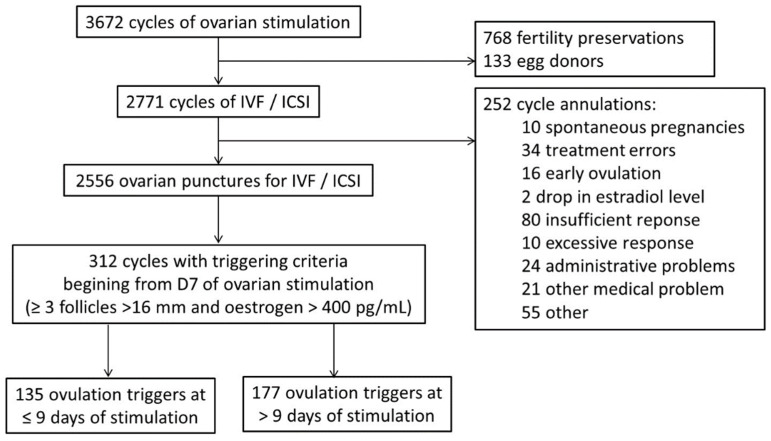
Flow chart.

Flow chart is presented in Figure 1.

Patient characteristics–age, body mass index (BMI), and ovarian reserve (antral follicle count and anti-Mullerian hormone (AMH) levels prior to stimulation) were systematically recorded. We also recorded the cause of infertility: endometriosis, diminished ovarian reserve (DOR), PCOS-associated dysovulation, tubal factors, and/or male infertility. Our analysis included the characteristics of the analysed IVF-ICSI cycle, i.e., fresh or frozen embryo transfers. Only the first live birth per patient was taken into consideration in the analysis.

All the women were informed that their data were routinely and prospectively entered into an electronic record-keeping system as part of the French national PMSI (‘Programme de médicalisation des systèmes d’information’) database, and that indicators were analysed. Therefore, informed consent was obtained from each subject before beginning ART. The data analysis protocol was validated by the local IRB (CEROG 2020-GYN-1205).

### 2.2. Procedure

Ovarian stimulation was conducted using either a GnRH-antagonist or GnRH-agonist protocol. The initial FSH dose ranged between 100 UL and 450 UL per day and was adapted to the patient’s BMI, age, and ovarian reserve. Ovulation was triggered by choriogonadotropin alfa (Ovitrelle, Merck Serono, Amsterdam, The Netherlands) or triptorelin 0.2 mg (Decapeptyl, IPSEN Pharma, Boulogne-Billancourt, France). Transvaginal oocyte retrieval was scheduled 36 h after triggering ovulation and IVF/ICSI was performed with fresh or cryopreserved sperm. Embryos were maintained in culture for 2 to 6 days and classified morphologically according to the Istanbul Consensus [8]. Fresh embryo transfer of one or two embryos was performed 2, 3, or 5 days after oocyte retrieval. The majority of embryos were transferred at blastocyst stage and the decision to transfer the embryo on day 2–3 was based on organizational factors and preferentially performed in women aged >38 years, with diminished ovarian reserve, <5 oocytes obtained at the puncture and the presence of top embryos at day 2 or 3. Luteal phase support included vaginal administration of 400 mg of progesterone daily starting from oocyte retrieval day and continued until the 12th week of gestation (WG) in the event of pregnancy.

Supernumerary embryos were cryopreserved on day 3 or 5 according to their quality. They were subsequently transferred in an artificial, natural, or stimulated cycle. The artificial cycle was obtained by a substitutive hormonal treatment consisting of 2 mg of oestradiol administered intravaginally (Provames, Sanofi-Aventis France, Luxembourg) and/or daily application of a 150 μg transcutaneous oestrogen patch (Vivelledot, Novartis, France) started on cycle day one or two. A pelvic ultrasound (US) scan was performed on day 10 of the cycle and progesterone was initiated if the endometrial thickness was >7 mm. In the natural cycle, ultrasound and blood monitoring were started on day 8 of the natural cycle and were repeated every 2–3 days until one follicle reached >16 mm or LH surge was observed. Progesterone was then started 2 days after LH surge or choriogonadotropin alfa (Ovitrelle, Merck Serono) administration. In stimulated cycles the initial dose of FSH ranged between 50–75 UI per day and the ovulation was triggered by choriogonadotropin alfa (Ovitrelle, Merck Serono, Amsterdam, The Netherlands) once at least one follicle reached 16 mm. Progesterone was started 2 days later. Embryo transfer was organized 2, 3, or 5 days of the progesterone administration depending on the age of cryopreserved embryo (D–2, D–3 and blastocyst, respectively).

Implantation was confirmed by positive serum human chorionic gonadotropin (hCG) assay 14 days after oocyte retrieval or 12 days after frozen embryo transfer. Clinical pregnancies were defined as detection of cardiac activity on pelvic US scan at 5 WG. The primary endpoint was live-birth rates defined as the percentage of live births over 23 WG.

### 2.3. Statistical Analysis

Descriptive analysis included frequencies and percentages for qualitative variables and mean with standard deviation for quantitative variables as suitable. The outcomes of all embryo transfers per patient were evaluated and cumulative live-birth rates were compared between women having had ovulation trigger on D ≤ 9 or D > 9 of ovarian stimulation. Only the first live birth per patient was taken into consideration in the analysis. Chi-square tests were used for qualitative variables and Student t-tests for quantitative variables. The predictive factors of a live birth were evaluated. A *p*-value < 0.05 was considered as statistically significant. Statistical analyses were performed using R studio software (version 1.3.1093, available online).

## 3. Results

### 3.1. Population Characteristics

During the study period, 312 women, corresponding to 12% of women triggered for IFV-ICSI, presented triggering criteria beginning from the eighth day (D8) of ovarian stimulation. Among them, 135 (43.3%) were triggered for ovulation before or on the 9th day of stimulation (D ≤ 9 group), and 177 (56.7%) after the ninth day of stimulation (D > 9 group). There was no statistical difference between the two groups in the choice of stimulation protocol (*p* = 0.263). Both groups were comparable in terms of age, BMI, and cause of infertility. The characteristics of the two groups are presented in Table 1.

### 3.2. IVF/ICSI Outcomes According to the Day of Ovulation Trigger

The mean oestradiol levels on D8 of stimulation were significantly higher in patients in the D ≤ 9 group (Table 2). The number of oocytes retrieved as well as the number of mature oocytes available for fecundation and the number of embryos obtained were significantly higher in the D > 9 group (10.8 ± 6.3 versus 13.5 ± 7.4, *p* = 0.001; 7.3 ± 3.7 versus 10.3 ± 5.0, *p* < 0.001; and 5.5 ± 4.1 versus 8.6 ± 5.6, *p* < 0.001, respectively). However, the clinical pregnancy rates, even if higher in D > 9 group, were not significantly different between the two groups (37% in the D ≤ 9 group versus 46.9% in the D > 9 group, *p* = 0.10). An oestradiol level >3500 pg/mL on the day of ovulation trigger was observed in 11 women in the D ≤ 9 group (8%) versus 18 (10%) in the D > 9 group (*p* = 0.54). The total live-birth rate after fresh and frozen embryo transfers was 24.4%. The live-birth rate was 19.3% in the D ≤ 9 group and 28.2% in the D > 9 group (*p* = 0.09).

The majority of embryos (67%) were transferred at blastocyst stage and there was no statistical difference in embryo transfer policy between both groups. There was no statistical difference in endometrial preparation protocols for frozen embryo transfer between the study groups (34 artificial, 11 natural, and 5 stimulated cycles in D ≤ 9 group versus 67 artificial, 20 natural, and 14 stimulated cycles in D > 9 group, *p* = 0.78).

### 3.3. Predictive Factors of Live Births in the D ≤ 9 Group

Among the patients in the D ≤ 9 group, age, BMI, initial AMH level, and cause of infertility, as well as the oestradiol level on D8 of the stimulation, were not predictive of the chances of a live birth. The number of oocytes retrieved, of mature oocytes available for fecundation, and the number of embryos available for transfer was statistically higher in women with a live birth (10.2 ± 6.2 versus 13.2 ± 6.4, *p* = 0.03; 6.8 ± 4.3 versus 9.4 ± 4.3, *p* = 0.03 and 5.0 ± 3.8 versus 7.7 ± 4.9, *p* = 0.01, respectively) (Table 3).

### 3.4. Predictive Factors of Live Births in the D > 9 Group

Patients who had a live birth in the D > 9 group were significantly younger and had a lower BMI than those without a live birth during the study period (33 ± 4.0 versus 35.2 ± 4.2, *p* = 0.001 and 23.5 ± 3.4 versus 24.9 ± 4.6, *p* = 0.03). However, initial AMH level, cause of infertility, and oestradiol levels on D8 of stimulation were similar in both groups. Even if the number of mature oocytes available for fecundation was higher in women who obtained a live birth (11.8 ± 9.7 versus 9.7 ± 5.2, *p* = 0.04), the number of embryos available for transfer was comparable between both groups (Table 4).

### 3.5. Patient Characteristics Associated with an Optimal Time for Ovulation Triggering

When comparing patients in the two groups who obtained a live birth during the study period, oestradiol levels on D8 of stimulation were significantly higher in patients in the D < 9 group (1848 ± 943 versus 1302 ± 693, *p* = 0.01) (Table 5). The cause of infertility and initial AMH level was not statistically significant in both groups (*p* = 0.86). The number of women who obtained a live birth with a low AMH level (defined as <0.5 ng/mL for patients aged 38 and older, and <1.0 ng/mL for patients aged 40 and older) was comparable between the D ≤ 9 group and the D > 9 group (*p* = 1). Age and BMI were not significantly associated with higher chances of success depending on the time of ovulation trigger.

Multivariate analysis was not performed as no significant difference was found between the study groups.

## 4. Discussion

In the present study analysing the IVF/ICSI results in women presenting ovulation trigger criteria beginning from D8 of the ovarian stimulation, no statistical differences in terms of clinical pregnancies and live births after fresh and frozen embryo transfers were observed, whether ovulation was triggered at D ≤ 9 or D > 9. No patient characteristics were found to be predictive of a live birth depending on the day of the ovulation trigger. However, oestradiol levels on D8 of stimulation were significantly higher in the D ≤ 9 triggered group compared to the women in the D > 9 triggered group.

Stimulation durations are known to affect oocyte maturation, embryo quality as well as endometrial receptivity [9,10]. Yoldemir et al. showed an increased rate of fragmentation and asymmetry of blastomeres for prolonged gonadotropin stimulations while Kolibianakis et al. showed an increase in endometrial advancement on the day of oocyte retrieval for prolonged follicular phases [11,12]. Prolonged ovarian stimulation can therefore decrease the survival of embryos at later stages after transfer while short stimulation durations may not allow enough time for endometrial development.

The relationship between stimulation durations and ovarian response has been documented with somewhat inconsistent findings. According to Yang et al., using the same criteria for oocyte maturation triggering, poor responders achieving a pregnancy seemed to have a shorter stimulation duration than normal responders achieving a pregnancy [9]. Prolonged stimulation durations, however, often correlate with poor ovarian response as it usually takes longer for these patients to achieve a sufficient number of follicles of acceptable size [13,14]. In the present study, we only included patients who achieved ovulation-triggering criteria at an early stage of gonadotropin stimulation, independently of their overall ovarian response.

One known parameter that affects stimulation duration independently of ovarian reserve is the type of stimulation protocol. The GnRH-antagonist protocol has been reported to have a shorter stimulation duration than the GnRH-agonist protocol without influencing pregnancy rates or live-birth rates [7]. In the present study, however, the number of GnRH-antagonist and agonist protocols used in each group were not significantly different.

A number of patient characteristics are known to influence ovarian stimulation outcomes. Among them, age, AMH levels, and causes of infertility are commonly used as predictors of IVF success [15,16]. In a recent study, Metello et al. showed a significant decrease in live-birth rates with increasing age while masculine-related infertility and ovulation disorders were significantly associated with higher chances of success [17]. In the present study of a cohort of women with early trigger criteria, no patient characteristics were found to be predictive of a live birth depending on the day of ovulation trigger. Our results suggest that while some characteristics may influence the patients’ chances of success, their prognosis remains unaltered once the criteria for triggering of ovulation are met during ovulation stimulation Therefore, these characteristics should not be taken into consideration when deciding the day of ovulation trigger. The stimulation trigger should thus be based on organisational criteria, and should take into account the woman’s comfort and the medico-economic aspects of ART.

Previous studies of the relationship between the number of oocytes retrieved and IVF outcomes have suggested that live-birth rates tend to increase up to a certain number of oocytes retrieved, ranging from 8 to 15, above which the rates tend to reach a plateau [18,19,20]. In our study, the number of oocytes retrieved was significantly higher in the D > 9 group with a mean of 13.5 versus 10.8 in the D ≤ 9 group (*p* = 0.001). However, the difference in the cumulative live-birth rates did not reach a statistical significance.

Oestradiol levels on D8 of stimulation were significantly higher in the D ≤ 9 group with a mean level of 872 pg/mL vs 692 pg/mL in the D > 9 group. The same difference was found when comparing women in the D ≤ 9 group who achieved a live birth compared to those in the D > 9 group. Those differences might be explained by the fact that most of the follicles present at day eight in the D ≤ 9 group were mature follicles and elevated estrogen levels might have reinforced the decision of the practitioner to trigger ovulation more rapidly. Although no threshold was found to be significant, these results suggest that patients with low oestradiol levels on D8 of stimulation may benefit from having their ovarian stimulation prolonged even if the criteria for ovulation trigger are met. However, further studies should be conducted to explore this hypothesis.

Live-birth rates found in recent studies range from 25% to 50% [15,17,21]. In our study, the total live-birth rate per IVF cycle with fresh and frozen embryo transfers was 24.4%–slightly lower than that found in literature–which could have limited statistical analyses. This discrepancy can be explained by the lower live-birth rate generally expected in women having non-optimal stimulation durations ( <9 days or >12 days), as found in a recent study by Sarka et al. [6]. Also, only one cycle of ovarian stimulation followed by fresh +/− frozen embryo transfers was analysed. For patients who had multiple live births during the study period, only the first live birth was counted in the analysis.

The major limitation of our study lies in its retrospective nature. However, our study was conducted in a relatively large cohort of women with similar characteristics in both groups. The difference in pregnancy and live-birth rates did not reach statistical significance, but in a group, with prolonged stimulation duration an increased number of mature oocytes has been observed with a trend toward higher pregnancy and live birth rates. However, we cannot exclude that for women who achieve triggering criteria by day eight there could be benefits to slightly prolong stimulation interval prior to triggering, which this study was underpowered to statistically confirm. Either way, the prolongation of the time to trigger ovulation did not seem to have a negative impact on IVF/ICSI issues. Further large prospective studies would be necessary to confirm our results.

The analysis of predictive factors of live birth did not allow identifying any clinical characteristics. However, this may be related to the limited sample size of the analysis.

To the best of our knowledge, our study is the first to address the issue of an optimal time to trigger ovulation for patients who achieve triggering criteria at an early stage of ovarian stimulation.

## 5. Conclusions

In conclusion, the time chosen to trigger ovulation did not seem to impact the live-birth rate or pregnancy outcomes for early responders once triggering criteria were met. A patient’s clinical characteristics did not impact the outcomes. This could point out that clinical parameters should not influence the decision on the day of ovulation trigger if the patient meets the triggering criteria beginning from day eight of ovarian stimulation. Our results suggest that it is reasonable to delay ovulation triggering if necessary for organisational purposes. However, further large prospective studies should be conducted to support our findings.

## Figures and Tables

**Table 1:** Characteristics of patients with ovulation trigger criteria present beginning from the eightth day of ovarian stimulation and with final ovulation trigger performed before or on the ninth day of stimulation (D ≤ 9) or after the ninth day of stimulation (D > 9).

	D ≤ 9 Group *n* = 135 (43.3%)	D > 9 Group *n* = 177 (56.7%)	*p*-Value
Age, years; mean (SD)	34.94 (4.48)	34.6 (4.22)	0.49
BMI kg/m^2^; mean (SD)	23.95 (3.99)	24.5 (4.31)	0.25
Type of infertility			0.28
Primary, *n* (%)	92 (68.1)	108 (61.0)	
Secondary, *n* (%)	43 (31.9)	67 (37.9)	
Cause of infertility			0.72
Endometriosis, *n* (%)	12 (8.9)	12 (6.8)	
DOR, *n* (%)	10 (7.4)	16 (9.0)	
Male factor, *n* (%)	37 (27.4)	56 (31.6)	
Mix/Other, *n* (%)	76 (56.3)	93 (52.5)	
AMH, ng/mL, mean (SD)	2.94 (2.41)	3 (2.03)	0.81
Stimulation protocol			0.26
GnRH-antagonist, *n* (%)	83 (61.5)	119 (67.2)	
Long GnRH-agonist, *n* (%)	39 (28.9)	49 (27.7)	
Short GnRH-agonist, *n* (%)	13 (9.6)	9 (5.1)	

AMH—Anti-Mullerian hormone. BMI—Body mass index. DOR—Diminished ovarian reserve. *p*-values were calculated with Student *t*-tests for quantitative variables and Chi square tests for qualitative variables.

**Table 2:** IVF/ICSI outcomes according to the day of ovulation trigger.

	D ≤ 9 Group *n* = 135 (43.3%)	D > 9 Group *n* = 177 (56.7%)	*p*-Value
E2 level on D8, pg/mL mean (SD)	1703 (872)	1434 (692)	0.004
E2 level > 1000 pg/mL on D8, *n* (%)	39 (28.9)	64 (36.2)	0.18
>3 follicles of 12–15 mm on D8, *n* (%)	73 (54.1)	142 (80.2)	0.27
Number of oocytes retrieved, mean (SD)	10.8 (6.3)	13.5 (7.4)	0.001
Number of mature oocytes, mean (SD)	7.3 (3.7)	10.3 (5.0)	<0.001
Number of transferable embryos, mean (SD)	1.9 (1.6)	3.0 (2.7)	<0.001
Number of frozen embryos, mean (SD)	0.9 (1.5)	2.0 (2.8)	<0.001
Fresh transfers with blastocyst, *n*/total fresh embryo transfers (%)	52/93 (56)	68/120 (57)	1
Frozen transfers with blastocyst, *n*/total frozen embryo transfers (%)	44/50 (88)	82/105 (78)	0.14
Number of clinical pregnancies after fresh and frozen embryo transfers, *n* (%)	50 (37.0)	83 (46.9)	0.10
Number of live births after fresh and frozen embryo transfers, *n* (%)	26 (19.3)	50 (28.2)	0.09

E2—oestradiol. *p*-values were calculated with Student *t*-tests for quantitative variables and Chi-square tests for qualitative variables.

**Table 3:** Predictive factors of live births when ovulation trigger occurs by D ≤ 9.

	D ≤ 9 without Live Birth *n* = 109	D ≤ 9 with Live Birth *n* = 26	*p*-Value
Age, years; mean (SD)	35.0 (4.6)	34.6 (4.1)	0.68
BMI, kg/m^2^; mean (SD)	23.96 (3.94)	23.87 (4.3)	0.13
Type of infertility			0.64
Primary, *n* (%)	73 (67.0)	19 (73.1)	
Secondary, *n* (%)	36 (33.0)	7 (26.9)	
Cause of infertility			0.55
Endometriosis, *n* (%)	10 (9.2)	2 (7.7)	
DOR, *n* (%)	8 (7.3)	2 (7.7)	
Male factor, *n* (%)	27 (24.8)	10 (38.5)	
Mix/Other, *n* (%)	64 (58.7)	12 (46.2)	
AMH ng/mL, mean (SD)	2.96 (2.56)	2.87 (1.73)	0.82
Low AMH level ^a^, *n* (%)	8 (7.3)	1 (3.8)	1.00
E2 level on D8, pg/mL mean (SD)	1669 (855)	1848 (943)	0.35
E2 level < 1000 pg/mL on D8, *n* (%)	31 (28.4)	8 (30.8)	0.81
Number of retrieved oocytes, mean (SD)	10.2 (6.2)	13.2 (6.4)	0.03
Number of mature oocytes, mean (SD)	6.8 (4.3)	9.4 (4.3)	0.03
Number of obtained embryos, mean (SD)	5.0 (3.8)	7.7 (4.9)	0.01

AMH—Anti-Mullerian hormone. BMI—Body mass index. DOR—Diminished ovarian reserve. E2—Oestradiol. ^a^ Low AMH: <0.5 ng/mL. *p*-values were calculated with Student *t*-tests for quantitative variables and Chi square tests for qualitative variables.

**Table 4:** Predictive factors of live births when ovulation trigger occurs at D > 9.

	D > 9 without Live Birth *n* = 127	D > 9 with Live Birth *n* = 50	*p*-Value
Age, years; mean (SD)	35.2 (4.2)	33.0 (4.0)	0.001
BMI, kg/m^2^; mean (SD)	24.9 (4.6)	23.5 (3.4)	0.03
Type of infertility			0.73
Primary, *n* (%)	76 (60.8)	32 (64)	
Secondary, *n* (%)	49 (39.2)	18 (36)	
Cause of infertility			0.06
Endometriosis, *n* (%)	6 (4.72)	6 (12)	
DOR, *n* (%)	14 (11.0)	2 (4)	
Male factor, *n* (%)	36 (28.3)	20 (40)	
Mix/Other, *n* (%)	71 (55.9)	22 (44)	
AMH ng/mL, mean (SD)	2.95 (2.14)	3.13 (1.74)	0.60
Low AMH level ^a^, *n* (%)	4 (3.1)	2 (4.0)	0.67
E2 level on D8, pg/mL mean (SD)	1487 (688)	1302 (693)	0.11
E2 level < 1000 pg/mL on D8, *n* (%)	40 (31.5)	24 (48.0)	0.055
Number of oocytes retrieved, mean (SD)	12.8 (7.6)	15.2 (6.6)	0.055
Number of mature oocytes, mean (SD)	9.7 (5.2)	11.8 (9.7)	0.04
Number of embryos obtained, mean (SD)	8.2 (5.8)	9.7 (4.8)	0.07

AMH—Anti-Mullerian hormone. BMI—Body mass index. DOR—Diminished ovarian reserve. E2—Oestradiol. ^a^ Low AMH: <0.5 ng/mL. *p*-values were calculated with Student *t*-tests for quantitative variables and Chi square tests for qualitative variables.

**Table 5:** Predictive factors of live births according to the day of ovulation trigger.

	D ≤ 9 Group with Live Birth *n* = 26	D > 9 Group with Live Birth *n* = 50	*p*-Value
Age, mean (SD)	34.6 (4.1)	33.0 (4.0)	0.1
BMI, mean (SD)	23.87 (4.30)	23.49 (3.35)	0.68
Type of infertility			0.45
Primary, *n* (%)	19 (73.1)	32 (64)	
Secondary, *n* (%)	7 (26.9)	18 (36)	
Cause of infertility			0.86
Endometriosis, *n* (%)	2 (7.7)	6 (12)	
IOP, *n* (%)	2 (7.7)	2 (4)	
Masculine, *n* (%)	10 (38.5)	20 (40)	
Mix/Other, *n* (%)	12 (46.2)	22 (44)	
AMH ng/mL, mean (SD)	2.87 (1.73)	3.13 (1.74)	0.53
Low AMH level ^a^, *n* (%)	1 (3.8)	2 (4.0)	1.00
E2 level on D8, pg/mL mean (SD)	1848 (943)	1302 (693)	0.01
E2 level < 1000 pg/mL on D8, *n* (%)	8 (30.8)	24 (48.0)	0.22

AMH—Anti-Mullerian hormone. BMI—Body mass index. DOR—Diminished ovarian reserve. E2—estradiol. ^a^ Low AMH: <0.5 ng/mL. *p*-values were calculated with Student *t*-tests for quantitative variables and Chi square tests for qualitative variables.

## Data Availability

The data presented in this study are available on request from the corresponding author.
